# Induction of the mesenchymal to epithelial transition by demethylation-activated microRNA-125b is involved in the anti-migration/invasion effects of arsenic trioxide on human chondrosarcoma

**DOI:** 10.1186/s13046-016-0407-y

**Published:** 2016-08-30

**Authors:** Xing Bao, Tingting Ren, Yi Huang, Shidong Wang, Fan Zhang, Kuisheng Liu, Bingxin Zheng, Wei Guo

**Affiliations:** 1 Musculoskeletal Tumor Center, Peking University People’s Hospital, No.11 Xizhimen South Street, Beijing, 100044 People’s Republic of China; 2 Beijing Key Laboratory of Musculoskeletal Tumor, Beijing, 100044 People’s Republic of China

**Keywords:** Arsenic trioxide (ATO), MicroRNA-125b, Chondrosarcoma, Signal transducer and activator of transcription3(Stat3), Metastatsis

## Abstract

**Background:**

In addition to treating acute promyelocytic leukemia, arsenic trioxide (ATO) suppresses other solid tumors, including chondrosarcoma. However, the effects of ATO on metastasis in chondrosarcoma cells, and the underlying molecular mechanisms remain unclear.

**Methods:**

The effects of ATO on the migratory and invasive capacities of chondrosarcoma cells were investigated by Wound healing, Transwell and EMT assays. The expression of miR-125b in human chondrosarcoma tissues and cell lines was detected by real-time PCR analysis. Bisulfite sequencing analysis (BSP) was used to detect the effects of ATO on the expression of miR-125b. The gain-of-function and loss-of-function experiments were performed on chondrosarcoma cell lines to investigate the effects of miR-125b on chondrosarcoma invasion, and to determine whether signal transducer and activator of transcription 3(Stat3) mediates these effects. Dual-luciferase reporter assay was used to identify whether Stat3 is a direct target of miR-125b.

**Results:**

MiR-125b was significantly downregulated in human metastatic chondrosarcoma tissues and cell lines but not in non-metastatic chondrosarcoma tissues. ATO up-regulates the expression of miR-125b by the demethylation of DNA. ATO induces MET and attenuates the invasive capacities of chondrosarcoma cells through miR-125b. Stat3 was verified as a direct target of miR-125b, which is involved in ATO regulating EMT-associated traits.

**Conclusions:**

These findings, for the first time, provides evidence that the miR-125b-mediated inhibition of Stat3 is involved in the ATO-induced attenuation of metastasis in chondrosarcoma cells.

## Background

Chondrosarcoma is the second most common primary bone tumor. Current practices for the treatment of chondrosarcoma include wide-margin surgical resection and chemotherapy and are less than satisfactory because of high recurrence rates and metastasis [1–3]. The mechanisms underlying metastasis and recurrence remain obscure. A continued search for molecular markers that predict metastasis and the recurrence of chondrosarcoma is essential.

Emerging evidence suggests that the epithelial-mesenchymal transition (EMT) contributes to tumor metastasis and recurrence in chondrosarcoma and other tumors [4, 5]. Chondrosarcoma cells undergoing EMT triggered by various stimuli might acquire altered traits involving chemoresistance, migration, and stemness [6, 7]. Although several lines of evidence have demonstrated the significance of the EMT in chondrosarcoma progression, the molecular mechanisms that regulate EMT remain unclear.

Accumulating evidence suggests that microRNAs (miRNAs) play important roles in regulating EMT in cancer cells [5, 8, 9]. MiRNAs, which are small, noncoding RNA molecules of 20–24 nucleotides, have the capacity to inhibit at the post-transcriptional and/or transcriptional level mainly by targeting the 3’-untranslated regions of mRNAs [10, 11].

It has been reported that almost every type of cancer, including chondrosarcoma, displays a specific profile of aberrantly expressed miRNAs that might be used as potential biomarkers or as therapeutic targets [12–18]. MiR-125b is a frequently downregulated miRNA in various types of tumors, including colorectal cancer and liver cancer [19, 20]. It has been reported that miR-125b is downregulated in human osteosarcoma cells [21]. However, there is little known about the effects of miR-125b in chondrosarcoma.

Arsenic trioxide (ATO) is an FDA-approved drug employed for the standard therapy of patients with acute promyelocytic leukemia (APL) [22]. In addition, the anti-cancer effects of ATO have been observed in other solid tumors, such as liver, breast, prostate and osteosarcoma [23, 24].

Some studies show that ATO treatment attenuates the EMT and invasive capacities of cancer cells [25, 26]. It has been reported that ATO could induce demethylation of DNA and cause up-regulation of certain genes and microRNAs (miRNAs) [27, 28].

Herein, we report that ATO attenuates the migratory and invasive capacities of chondrosarcoma cells in vitro and in vivo. In human chondrosarcoma cells, ATO up-regulates the expression of miR-125b by the demethylation of DNA, which induces the MET process in vitro and in vivo.

Furthermore, we demonstrated that miR-125b inhibited the EMT process in chondrosarcoma cells by targeting signal transducer and activator of transcription 3 (Stat3); constitutive activation of Stat3 is a key player in tumor angiogenesis and metastasis.

We believe that the data from our study could provide a new mechanism underlying the anti-migration/invasion effect of arsenic trioxide on human chondrosarcoma.

## Methods

### Human tissue specimen

Fourteen chondrosarcoma tissues from the patients with metastases and non-metastases, 7 samples from the adjacent normal tissues (located > 3 cm away from the tumor) were collected under the protocols approved by the ethics committee of Peking University People’s Hospital. Informed consents (written in the light of the ethical guidelines) were obtained from all the patients. The clinical characteristics of these patients were shown in Table 1. Fresh tissues were stored in liquid nitrogen before RNA extraction. The paraffin-embedded pathological specimens from 10 patients with chondrosarcoma and adjacent nontumor tissues were obtained from the Department of Pathology and the Musculoskeletal Tumor Center, Peking University People’s Hospital (Beijing, China).

**Table 1:** Clinical characteristics of patients with chondrosarcoma

Parameter	Cases
Age (years)	
≤ 18	8
> 18	6
Gender	
Male	9
Female	5
Sties	
Humerus	3
Femur	6
Tibia	3
Other	2
Metastasis	
Present	7
Absent	7

### Cell culture and reagents

The human articular chondrocyte cell line HC-a (Sciencell, Carlsbad, CA, USA) was maintained in DMEM supplemented with 15 % fetal bovine serum, plus antibiotics. SW1353 cells were obtained from American Type Culture Collection (ATCC, Manassas, VA) and were maintained in L-15 medium (Gibco, Grand Island, NY). OUMS-27 cells, HCS-2/8 cells and JJ012 cells were kindly gifted from Dr. J Block (Rush Medical College, Chicago, IL, USA) and were cultured in Dulbecco Modified Eagle Medium (Hyclone, Logan, UT) supplemented with 10 % fetal calf serum (Gibco, Grand Island, NY) at 37 °C in a humidified atmosphere with 5 % CO2.

ATO was purchased from Sigma Chemical Co. (St. Louis, MO, USA). The following antibodies were used in the experiments: anti-p-STAT3, anti-STAT3, anti-E-cadherin, anti-Vimentin, anti-Bax and anti-GAPDH were from Cell Signaling Technology (Beverly, MA, USA). anti-N-cadherin, anti-Slug, anti-MMP9 were from Abcam (USA). STAT3 siRNA were purchased from Suzhou GenePharma (Suzhou, China). Lipofectamin 3000 was bought from Origene (Rockville, MD, USA).

### Western blot analysis

Equal amounts of proteins collected from different kinds of cell lysates were loaded on 10–15 % SDS-PAGE gels using a NuPAGE system (Invitrogen) and then transferred onto PVDF membranes as previously described [22].

### Immunohistochemistry

Paraffin sections were reacted with rabbit polyclonal anti-p-Stat3, anti-vimentin, anti-N-cadherin and anti-E-cadherin antibodies (1:200 dilution). Sections stained with nonimmune rabbit serum (1:200 dilution) in phosphate-buffered saline (PBS) instead of primary antibody served as negative controls. Cells exhibiting positive staining on cell membranes and in the cytoplasm and nucleus were counted in at least 10 representative fields (×400magnification) and the mean percentage of positive cells was calculated. Immunostaining was assessed by two independent pathologists blinded to clinical characteristics and outcomes.

### Wound healing assay

A total of 2 × 10^5^ OUMS-27, HCS-2/8 and SW1353 cells were seeded into a 24-well plate. The tumor cells were grown to confluence 24 h later. An artificial wound was introduced with a P10 pipette tip in each well. The data of the wounded area were recorded at 0 h and 24 h with a microscope (Olympus Corp). The entire assay was repeated three times.

### Transwell assay

OUMS-27 and SW1353 cells were harvested, washed, suspended with RPMI1640 (GIBCO, Life Technologies, 11965–092) or L-15 medium (Gibco, Grand Island, NY), and seeded into the upper chambers of transwell inserts (8 μ m pore size; Corning) with or without 1.5 μM ATO in the migration assay. The upper chambers were coated with Matrigel (BD Bioscience, 354234) before the inoculation of the cancer cells and ATO in the invasion assay.

The lower compartments were filled with RPMI1640 or L-15 medium supplemented with 5 % FBS (fetal bovine serum). The cells in the upper chamber were removed with a swab after incubation for 12 h in the migration assay or 24 h in the invasion assay. The cells that migrated to the lower layer and attached to the membrane were stained with crystal violet and were counted in five fields per well under a microscope. The whole assay was repeated three times.

### Epithelial-mesenchymal transition (EMT) induction

OUMS-27 and HCS-2/8 cells were cultured to attach in complete medium overnight. Then, the cells were maintained in either medium alone or medium supplemented with 2.5 ng/ml TGF-β 1 (R&D Systems, Minneapolis, MN) with or without 1.5 μM 5 ATO in a humidified 5 % CO_2_ incubator at 37 °C. The morphologic photos of the cells were taken seven days after the incubation. The experiments were repeated three times.

### Quantitative RT-PCR (qRT-PCR)

The miRNAs were isolated from chondrosarcoma tissues or cell lines using an RNeasy/miRNeasy Mini kit (Qiagen, Limburg, The Netherlands) according to the manufacturer’s instructions. Total RNA was isolated using the TRIzol reagent (Invitrogen). The cDNAs were synthesized using a RevertAidTM First Strand cDNA Synthesis kit (Fermentas, Vilnius, Lithuania), and real-time quantitative PCR was carried out using the SYBR-Green PCR Master Mix (Applied Biosystems, Foster City, CA, USA) on a 7900 Real-Time PCR System (Applied Biosystems).

The primers used in this study were as follows: STAT3-F, 5′- CCCCATACCTGAAGACCAAG-3′and STAT3-R, 5′- GGACTCAAACTGCCCTCCT-3′; E-cadherin, CDH-1-F, 5′- TGCTCACATTTCCCAACTC-3′ and CDH-1-R, 5′-TCTGTCACCTTCAGCCATC-3’; N-cadherin, CDH-2-F, 5′- CTGACAATGACCCCACAGC-3′and CDH-2-R, 5′-TCCTGCTCACCACCACTACTT-3′; miR-125b, 5′-AATCCCTGAGACCCTAACTTGTGA-3′.

### MiR-125b target prediction and cell transfection

Three online programs, PicTar (http://pictar.mdc-berlin.de), and Tarbase (http://diana.cslab.ece.ntua.gr/tarbase), were used in combination with previous reports for predicting the target genes of miR-125b.

Anti-miR-125b and anti-con lentivirus vector construction, lentivirus packaging, and cell transfection have been previously described [20]. SiStat3, miR-125b-mimic, miR-125b-inhibitor, and miRNA-NC (negative control) were synthesized by Suzhou GenePharma. Cells were transiently transfected using Lipofectamine 2000 reagent (Invitrogen) for 12 h, according to the manufacturer’s protocol.

### Luciferase reporter assay

The Stat3 3′-untranslated region (3′UTR) containing the wild type or mutated miR-125b binding sequences were synthesized by Genescript (Nanjing, Jiangsu, China), and were cloned into the pmirGLO luciferase reporter vector (Promega, Madison, WI, USA). HEK293 cells were transfected with the wild type/mutant Stat3 luciferase reporter vector and miR-125b mimic/miR-Control using Lipofectamine 2000. Firefly and Renilla luciferase activities were measured using the Dual-Luciferase Reporter Assay System (Promega). Results were expressed as the firefly luciferase activity normalized to Renilla luciferase activity.

### Bisulfite sequencing analysis (BSP)

The methylation status of miR-125b promoter was determined by BSP. miR-125b DNA was extracted using a DNA kit (Qiagen 51306, Germany), and 2 μg of DNA was subjected to bisulfite conversion using an EpiTect Bisulfite Kit (59104, Qiagen, Germany) according to the manufacturer’s instructions. The transformed DNA was then PCR-amplified using the TaKaRa rTaq Kit (R001B, TaKaRa, Dalian, China). The PCR amplification products were sequenced by Invitrogen Corporation, Shanghai.

The primers for miR-125b, miR-125b-F, 5’-TTTATTTTTAGTTTGATGAGGAAAG -3’, and miR-1125b-R, 5’-CACCAAACTATCATTTAATAAACAC -3’ were used for qRT-PCR.

### Generation of xenografts

Six-week-old BALB/c female athymic nude mice (Vitalriver, Beijing, China) were subcutaneously injected in the right flank with cells (2 × 10^6^ in 0.1 ml PBS). After chondrosarcoma cells developed palpable tumors, the mice were randomly divided into two groups and administered intraperitoneally with DMSO or ATO at a dose of 2 mg/kg every other day for 30 days. The volume of xenografts was measured every five days (tumor volume = (length × width^2^)/2). The mice were sacrificed after 30 days. Tumor samples were processed for routine IHC.

The tumor metastatic ability of HCS-2/8 cells (5 × 10^6^cells) was observed following cell injection intravenously into the tail vein. Four weeks later, the mice were randomly divided into two groups and administered intraperitoneally with DMSO or ATO at a dose of 2 mg/kg every other day for 30 days (*n* = 6 per group); the mice were then sacrificed, and the number of metastatic nodules on the lung surface was counted. Metastatic lungs were fixed with 4 % paraformaldehyde before dehydration and paraffin embedding. Paraffin sections were stained with hematoxylin and eosin according to standard protocols.

### Statistical analysis

The influence of ATO on the migration, invasion, EMT and tumor formation of chondrosarcoma cells was analyzed by Student *t* test or One-Way ANOVA. In all statistical analyses, statistical significance in the two-sided test was indicated with P values of 0.05 or less, and a P value less than 0.01 was remarkably significant.

## Results

### MiR-125b expression was downregulated in human metastatic chondrosarcoma tissues and cells

**Fig. 1:**
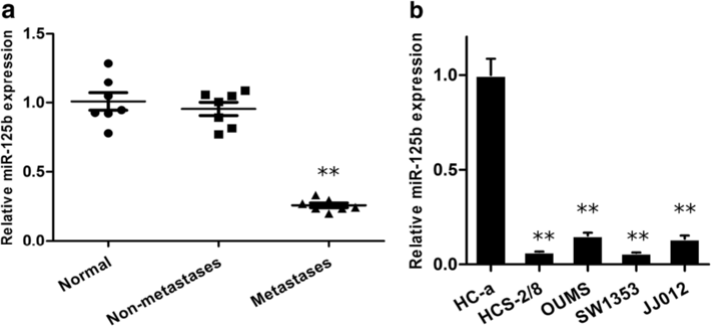
Expression of miR-125b in human chondrosarcoma tissues and cells. **a** Relative expression of miR-125b in chondrosarcoma tissues from the patients with metastases, non-metastases and the adjacent normal tissues. **P < 0.01 compared with normal. **b** Fold change of miR-125b expression in human chondrosarcoma cell lines (HCS-2/8, OUMS-27, SW1353, and JJ012) and the normal human articular chondrocyte cell line (HC-a). **P < 0.01 compared with HC-a

The expression level of miR-125b was quantified by realtime quantitative reverse transcription PCR in primary chondrosarcoma tissues and chondrosarcoma cell lines. The results showed that there is no significant difference between the adjacent normal tissues and the non-metastatic chondrosarcoma tissues in the expression level of miR-125b. However, miR-125b expression was significantly lower in metastatic chondrosarcoma tissues than that in adjacent normal tissues and non-metastatic chondrosarcoma tissues (Fig. 1a, **P < 0.01). Compared with the human articular chondrocyte cell line HC-a, the expression levels of miR-125b were significantly downregulated in the chondrosarcoma cell lines, including HCS-2/8, OUMS-27, SW1353, and JJ012 (Fig. 1b, **P < 0.01).

### ATO attenuates the migratory and invasive capacities of chondrosarcoma cells

**Fig. 2:**
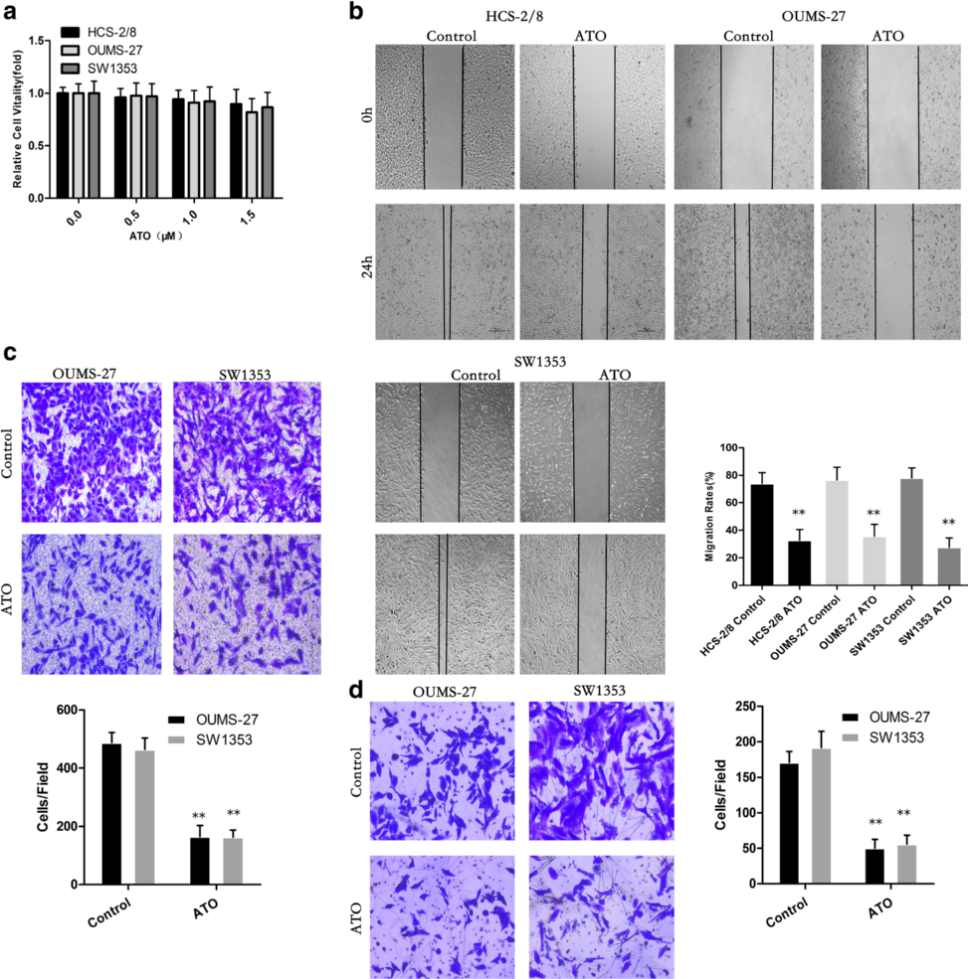
ATO Attenuates the Migratory and Invasive Capacities of Chondrosarcoma Cells. **a** ATO did not appreciably affect the vitality of HCS-2/8, OUMS-27 or SW1353 cells at concentrations of 0.5, 1.0 or 1.5 μM. **b** ATO reduces the migration of chondrosarcoma cells in wound-healing assays. Migration rates were calculated by the healing area/wound area after 24 h. **c** ATO significantly suppresses the migration of chondrosarcoma cells with transwell analysis. **d** ATO significantly inhibits the invasion of chondrosarcoma cells with Boyden chamber analysis. (*P < 0.05, **P < 0.01)

ATO did not appreciably affect the vitality of HCS-2/8, OUMS-27 or SW1353 cells at concentrations of 0.5, 1.0 or 1.5 μM (Fig. 2a).

HCS-2/8, OUMS-27 and SW1353 cells displayed high migration and invasion capacities, although ATO attenuated migration, as determined by wound healing assays, at a concentration of 1.5 μM (Fig. 2b). This concentration was selected as the maximum for further investigations. Furthermore, as determined with transwell assays, ATO decreased the invasive capacity of HCS-2/8, OUMS-27 and SW1353 cells (Fig. 2c and d). These results show that ATO could effectively attenuate the migratory and invasive capacities of human chondrosarcoma cells.

### ATO suppresses the epithelial-mesenchymal transition of chondrosarcoma cells

**Fig. 3:**
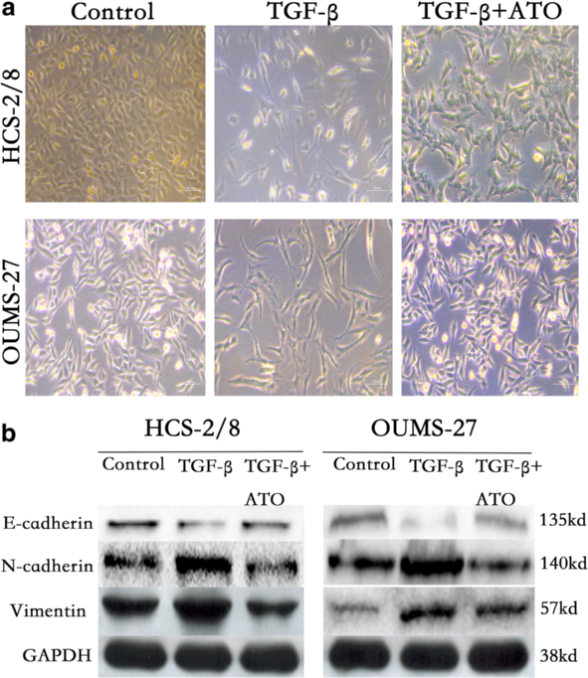
ATO suppresses the epithelial-mesenchymal transition of chondrosarcoma cells. **a** ATO reverses the EMT transition induced by TGF-β1 in chondrosarcoma cells. **b** Protein expression levels of Vimentin, N-cadherin and E-cadherin were determined in HCS-2/8 and OUMS-27 cells with TGF-β1 and ATO treatment

The epithelial-mesenchymal transition (EMT) is a critical process for epithelial cells to harbor mesenchymal properties and is closely involved in cancer invasion and metastasis [18]. To evaluate the effect of ATO on this process, we induced EMT of chondrosarcoma cells with TGF-β1 [18, 19] and observed that the cell lines showed a mesenchymal appearance, which was fibroblast-like (Fig. 3a). When treated with ATO, however, the chondrosarcoma cell lines maintained their epithelial appearance, indicating that ATO efficiently blocked the EMT induced by TGF-β1. Moreover, ATO impaired the expression of mesenchymal cell markers, including N-cadherin, Vimentin and slug; however, there was increased epithelial cell marker E-cadherin (Fig. 3b).

### ATO up-regulates the expression of miR-125b by the demethylation of DNA in HCS-2/8 and OUMS-27 cells

**Fig. 4:**
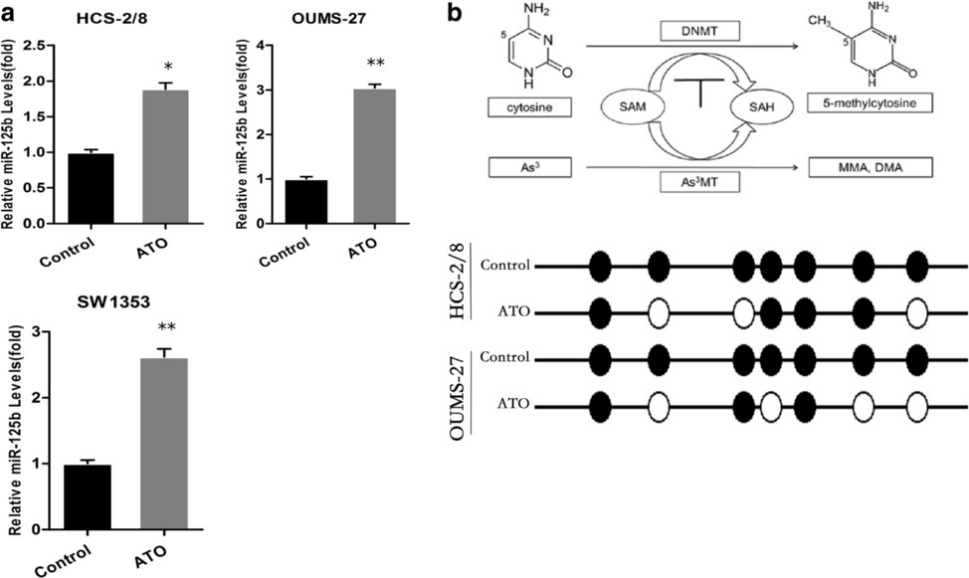
ATO Up-Regulates the Expression of miR-125b by Demethylation of DNA in Chondrosarcoma Cells. **a** HCS-2/8, OUMS-27 and SW1353 cells were exposed to 1.5 μM ATO for 24 h. qRT-PCR analyses of miR-125b levels (mean ± SD, *n* = 3). **P < 0.01 compared with control cells. **b** A pathway showing the demethylation effects induced by As^3+^. As^3+^MT (As^3+^ methyltransferase), MMA, monomethylarsinic acid; DMA, dimethylarsinic acid. **c** BSP shows that the miR-125b methylation ratio of the 7 CpG loci became 57.1 % (4/7) and 42.8 % (4/7) from 100.0 % in HCS-2/8 and OUMS-27 cells, respectively

As demonstrated herein, in ATO-treated HCS-2/8, OUMS-27 and SW1353 cells, there was increased expression of miR-125b. The qRT-PCR results for miR-125b expression in the experiments are shown (Fig. 4a).

Biotransformation of arsenic results in a deficiency of methyl donors, reducing DNA methylation, which activates a series of target genes [29]. We hypothesized that ATO up-regulated the expression of miR-125b by demethylation.

BSP was used to evaluate the promoter methylation status of miR-125b.

HCS-2/8 and OUMS-27 cells were treated with 0 or 1.5 μM of ATO. The methylation ratio of the 7 CpG loci became 57.1 % (4/7) and 42.8 % (4/7) from 100.0 %, respectively (Fig. 4b).

### ATO induces MET and attenuates the invasive capacities of chondrosarcoma cells through miR-125b

**Fig. 5:**
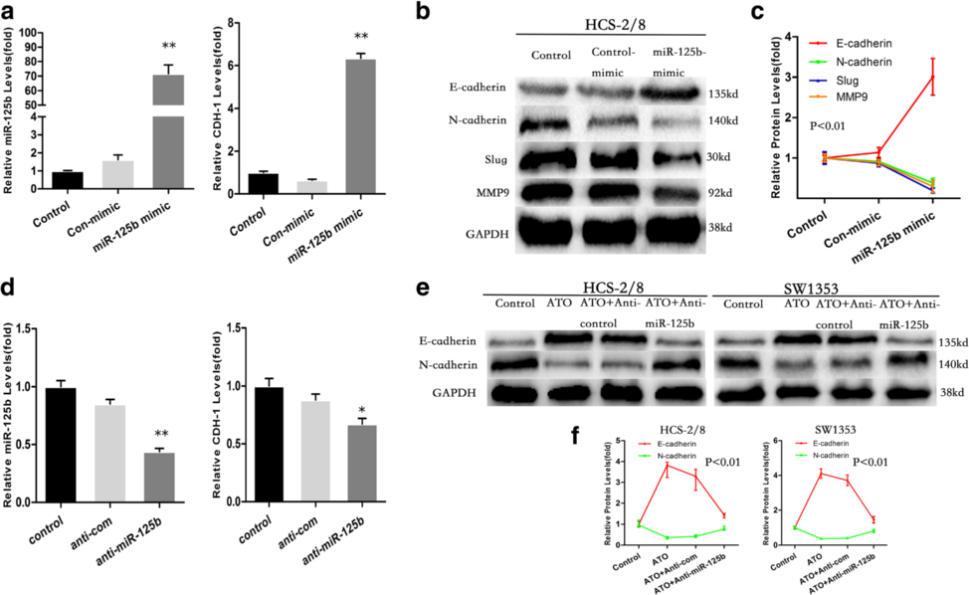
ATO induces MET and attenuates the invasive capacities of chondrosarcoma cells through miR-125b. HCS-2/8 cells were transfected with a con-mimic or miR-125b-mimic for 12 h. **a** qRT-PCR analyses of miR-125b and CDH-1 levels (mean ± SD, *n* = 3). **b** Western blot analyses and (**c**) relative protein levels of E-cadherin, N-cadherin, Slug and MMP9 (mean ± SD, *n* = 3). **P < 0.01 and *P < 0.05 compared with control cells or cells transfected with con-mimic. After HCS-2/8 cells were transfected by anti-con or anti-miR-125b for 12 h, they were exposed to 1.5 μM ATO for 48 h. **d** qRT-PCR analyses of miR-125b and CDH-1 levels (mean ± SD, *n* = 3); **e** Western blot analyses and (**f**) relative protein levels of E-cadherin and N-cadherin (mean ± SD, *n* = 3)

The present data show that the over-expression of miR-125b enhanced the expression of E-cadherin but decreased the expression of N-cadherin, Slug and MMP9 in HCS-2/8 cells (Fig. 5a–c). After HCS-2/8 cells were transfected with anti-con or anti-miR-125b for 12 h, the cells were exposed to 0.0 or 1.5 μM ATO for 48 h. The ATO-induced increased expression of E-cadherin and decreased expression of N-cadherin were impaired by knockdown of miR-125b (Fig. 5 d–f).

**Fig. 6:**
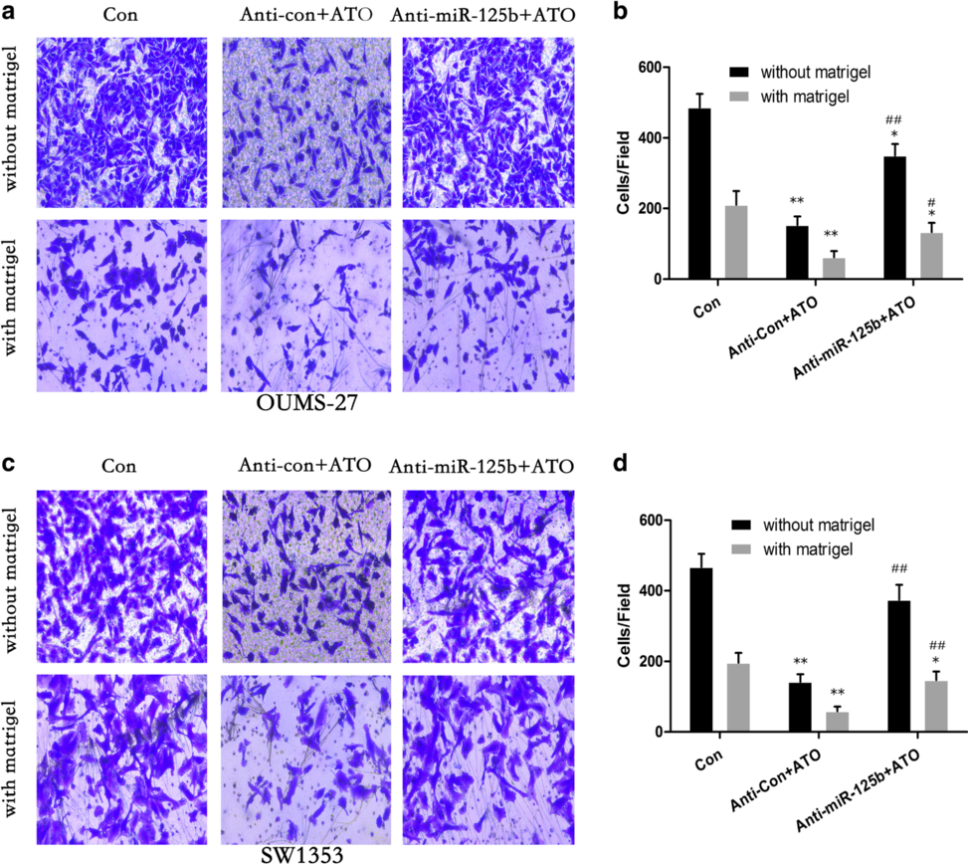
After OUMS-27 and SW1353 cells were transfected by anti-con or anti-miR-125b for 12 h, they were exposed to 1.5 μM ATO for 48 h. Results of transwell assays and relative levels of cell invasive capacity (mean ± SD, *n* = 3) in OUMS-27 (**a** and **b**) and SW1353 (**c** and **d**) cells. **P < 0.01 compared with medium control cells; ^#^ *P* < 0.05 and ^##^ *P* < 0.01 compared with anti-con-transfected cells treated by ATO

ATO attenuated the invasive capacity, as determined by transwell assays, in chondrosarcoma cells; however, the knockdown of miR-125b reduced these effects in chondrosarcoma cells (Fig. 6).

These results indicate that in HCS-2/8, OUMS-27 and SW1353 cells, miR-125b is involved in the ATO-induced MET and the inhibition of invasive capacity.

### Stat3 is a novel direct target of miR-125b, which is involved in ATO regulating EMT-associated traits

We explored the molecular mechanisms by which miR-125b affects ATO-mediated EMT suppression.

Bioinformatics analysis using PicTar (http://pictar.mdc-berlin.de) indicated the potential binding sites in the 3’UTR of Sat3 (Fig. 6g). Previous studies suggested that Stat3 is a significant inducer of EMT by the transcriptional activation of slug and twist1 [30].

**Fig. 7:**
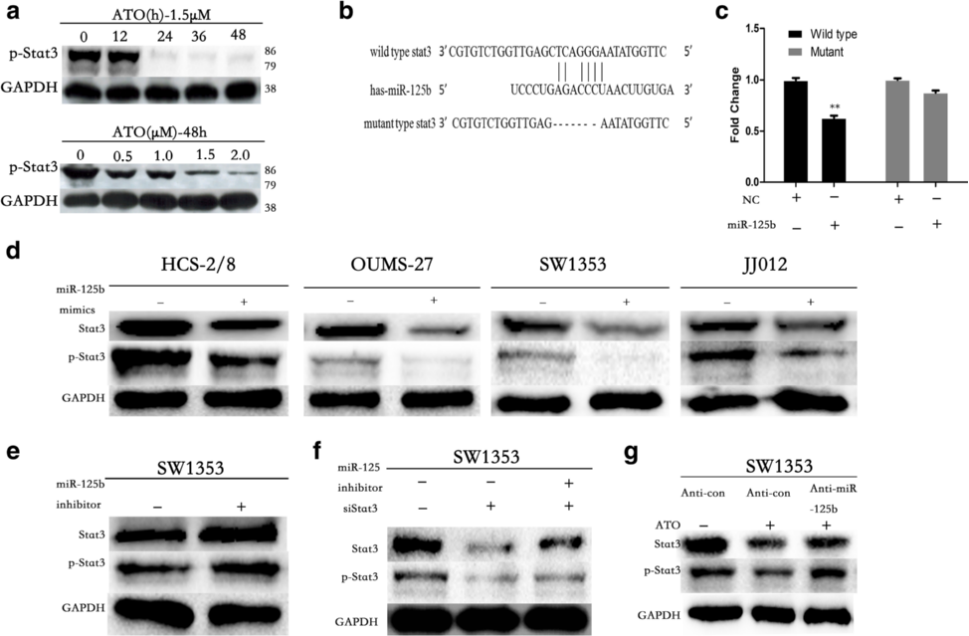
Stat3 is a novel direct target of miR-125b, which is involved in ATO regulating EMT-Associated traits in chondrosarcoma cells. **a** ATO inhibits Stat3 in a time and dose-dependent manner. **b** The Stat3 3′UTR region containing the wild type or mutant binding site for miR-125b. **c** Dual-luciferase reporter assay of miR-125b with 3’UTR fragments (wild type or mutant) of human Stat3 (NM_003150.3). Western blotting analysis of Stat3 expression in miR-125b-overexpressing cells (**d**) and in cells post-miR-125b inhibitor transfection (**e**) or with cotransfection with si-Stat3 (**f**). **g** Knockdown of miR-125b blocked the ATO-induced inactivation of Stat3 in HCS-2/8 and OUMS-27 cells

We examined the effects of ATO on the activation of Stat3 in chondrosarcoma cells. As shown in Fig. 7a, ATO decreased the activation of Stat3 in a time and dose-dependent manner in HCS-2/8 cells.

We detected a direct binding of miR-125b to the 3’UTR of Stat3 with a dual-luciferase reporter assay (Fig. 7b and c) and observed significant downregulation of Stat3 in miR-125b-overexpressing chondrosarcoma cells (Fig. 7d). We also found that miR-125b knockdown up-regulated Stat3 expression and rescued siRNA-mediated Stat3 suppression in chondrosarcoma cells (Fig. 7e and f). The knockdown of miR-125b blocked the ATO-induced inactivation of Stat3 in HCS-2/8 and OUMS-27 cells (Fig. 7g).

### ATO up-regulates the expression of miR-125b and inhibits metastasis in vivo

**Fig. 8:**
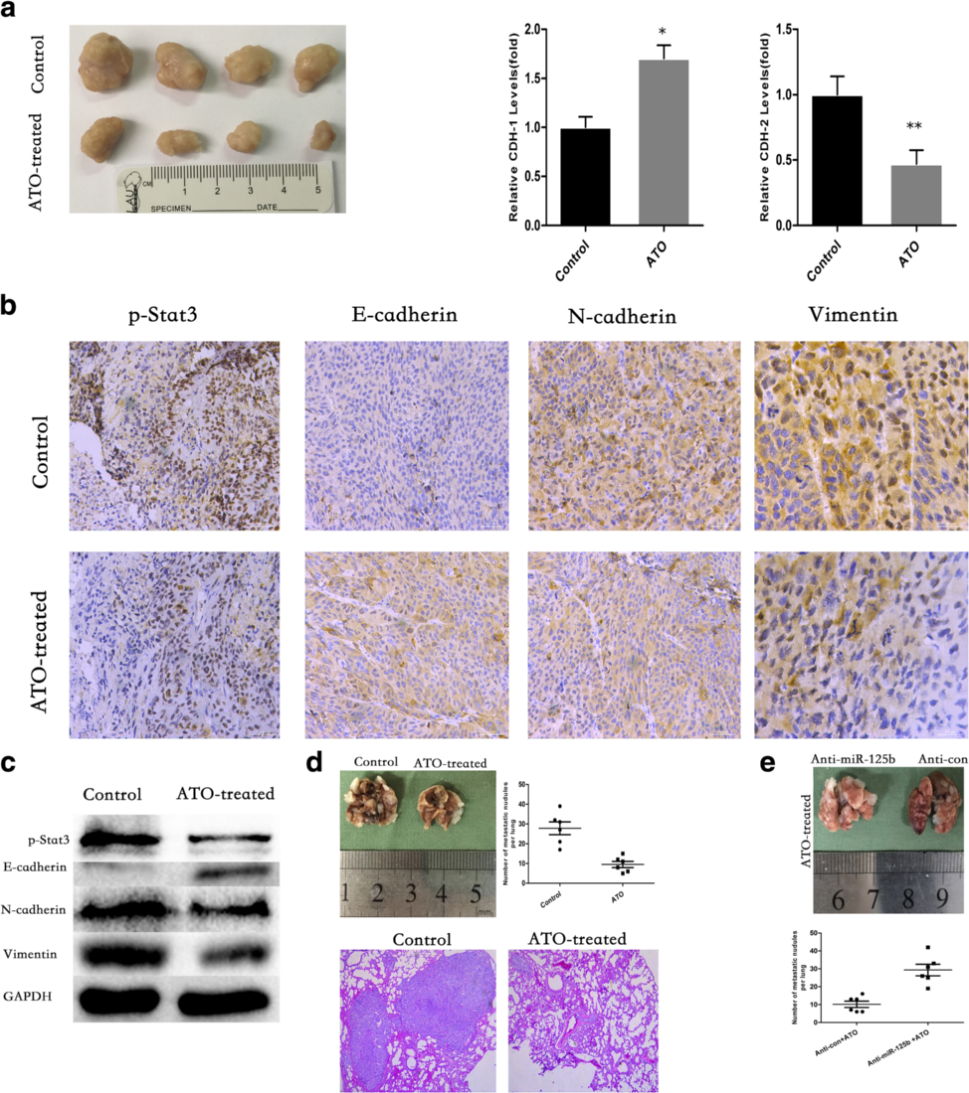
ATO Up-Regulates the Expression of miR-125b and Inhibits Metastasis In Vivo. **a** Observation of tumors formed by HCS-2/8 cells (left). qRT-PCR analyses of miR-125b, CDH-1 and CDH-2 levels in xenografts (Right) (mean ± SD, *n* = 3). **P < 0.01 compared with tumors treated with no ATO. **b** Immunohistochemistry analyses of p-Stat3, E-cadherin, N-cadherin and vimentin levels. **c** Western blot analyses of p-Stat3, E-cadherin, N-cadherin and vimentin. **d** Number of metastatic nodules on the surface of the lungs of mice injected with DMSO or ATO is presented. Representative images and H&E staining of lungs on day 70 after the mice were injected with HCS-2/8 cells (*n* = 6 per group). **e** Number of metastatic nodules on the surface of the lungs of mice formed by stable anti-miR-125b or anti-con HCS-2/8 cells is presented

Five days after HCS-2/8 cells were injected subcutaneously into the right armpit of nude BALB/c mice, the mice were randomly divided into two groups, respectively. Sterile saline or ATO (2 mg/kg BW) was administered intraperitoneally in 100 mL of sterile saline every other day for another 4 weeks. At that time, the tumors were removed and evaluated by qRT-PCR, immunohistochemistry and Western blot. In line with the in vitro data, ATO increased the E-cadherin expression and decreased the p-Stat3, vimentin and N-cadherin expression in tumors formed by chondrosarcoma cells (Fig. 8a–c).

ATO also markedly attenuated the metastatic potential of chondrosarcoma cells, as evidenced by the decreased lung metastasis occurrence. After HCS-2/8 cells were injected intravenously into the tail vein at 2 weeks, the mice were intraperitoneally administered ATO (0 or 2 mg/kg BW) in 100 mL of sterile saline every other day for another 30 days. The ATO-treated mice displayed statistically significantly lower numbers of lung metastases than the control group. After hematoxylin and eosin staining, an observation of the lungs showed that fewer lung metastatic nodes were observed in the mice treated with ATO (Fig. 8d).

To confirm the function of miR-125b in ATO-induced suppression of EMT, we established stable anti-miR-125b and anti-con HCS-2/8 cell lines. After the two stable cell lines were injected intravenously into the tail vein at 2-weeks, the mice were treated as previously described. Fewer lung metastatic nodules were observed in the anti-con group compared with the anti-miR-125b group (Fig. 8e).

Together, these findings strongly indicate that ATO inhibits EMT and invasive capacity through the miR-125b/Stat3 axis in chondrosarcoma cells.

## Discussion

Chondrosarcoma is the second most frequent type of primary bone cancer following osteosarcoma [1–3]. At present, surgical resection remains the only effective means of treating chondrosarcoma; however, the prognosis of chondrosarcoma remains poor because of high resistance to conventional chemo- and radiotherapy and metastasis. For this reason, anti-metastasis therapy has become the new way for the treatment of chondrosarcoma [29].

EMT, consisting in the transdifferentiation of epithelial cells into mesenchymal cells, is closely related to the regulation of immune and chemo resistance, the cancer stem cell phenotype and metastasis in cancer [30, 31]. Accumulating evidence suggests that Snail1, Twist1, Slug and Zeb1 are crucial to EMT, which reflected in repression of epithelial markers like E-cadherin and upregulation of mesenchymal markers like vimentin, N-cadherin and MMPs [32].

ATO is effective in treating acute promyelocytic leukemia, and its therapeutic effects on solid tumors are being evaluated [33]. In vivo studies, a dose range from 0.5 to 5.0 mg/kg of ATO and the i.p. route were used in the mouse model to determine the effects of arsenic trioxide on the EMT, metastasis, invasion, and angiogenesis of several cancers [34–37].

Our previous study showed that ATO treatment led to the promotion of G2/M arrest and the induction of apoptosis and autophagy in chondrosarcoma cells [22]. However, little is known regarding the effects of ATO on the EMT and metastasis of human chondrosarcoma and the underlying molecular mechanisms.

Herein, we exposed chondrosarcoma cells to 2 μM ATO and found that ATO inhibites EMT and attenuates the migration and invasion of chondrosarcoma cells. Meanwhile, there was no detectable cytotoxicity of 2 μM ATO in chondrosarcoma or the human articular chondrocyte cell line HC-a. This suggests that arsenic trioxide plays a positive effect in inhibiting metastasis in chondrosarcoma. However, the mechanism about inhibition of EMT and metastasis by ATO is obscure in chondrosarcoma.

It is reported that miRNAs play significant role in EMT [38]. MiRNAs are small non-coding RNAs that regulate gene expression by binding to the 3’-UTR of the target mRNA and inhibiting translation or targeting the mRNA for degradation [12]. Almost every type of cancer, including chondrosarcoma, displays a specific profile of aberrantly expressed miRNAs [39–48]. MiR-125b is reported to be a tumor suppressor gene in HCC, ovarian cancer, bladder cancer, breast cancer and osteosarcoma [19–21]. The roles of miR-125b in chondrosarcoma and the underlying molecular mechanisms remain largely uninvestigated.

Several studies reveal that ATO affects the tumor cell growth and metastasis through a complicated process that includes the regulation of signal pathways, transcriptional factors and microRNA [29, 35, 49].

Herein, in ATO-treated chondrosarcoma cells, for the first time, we identified that miR-125b exhibited a tumor suppressor function, which attenuated the EMT and invasive capacities by targeting Stat3, which is activated to trigger the molecular events in the EMT of various human tumors [50–54]. Furthermore, our data revealed that the elevation of miR-125b by ATO was dependent on the DNA demethylation. This study suggests a promising therapeutics target in chondrosarcoma and probably in other types of metastatic malignant tumors.

## Conclusions

The present study, for the first time, provides evidence that the miR-125b-mediated inhibition of Stat3 is involved in the ATO-induced attenuation of metastasis in chondrosarcoma cells.

## Abbreviations

ATO: Arsenic trioxide
EMT: Epithelial-mesenchymal transition
IHC: Immunohistochemistry
miRNA or miR: microRNA
siRNA: Small interfering
Stat3: The transcription factor signal transducer and activator of transcription 3
